# Antimicrobial Susceptibility and Apparent Multidrug Resistance Screening Patterns of Bacterial Pathogens Recovered from Clinical Samples of Companion Animals in Jordan, 2012–2024: A Retrospective Study from a Veterinary Teaching Hospital

**DOI:** 10.3390/vetsci13090892

**Published:** 2026-08-31

**Authors:** Mays Malkawi, Mousa Daradka, Zuhair Bani Ismail

**Affiliations:** Faculty of Veterinary Medicine, Jordan University of Science and Technology, Irbid 22110, Jordan; daradka@just.edu.jo (M.D.); zuhair72@just.edu.jo (Z.B.I.)

**Keywords:** antimicrobial resistance, multidrug-resistant, companion animals, dogs, cats, bacterial pathogens, antimicrobial susceptibility testing, Jordan, antimicrobial stewardship

## Abstract

Antimicrobial resistance is an increasing concern in veterinary medicine and within the One Health framework, yet long-term data from companion animals remain limited in Jordan. This retrospective study characterized bacterial pathogens and archived antimicrobial susceptibility patterns among clinical samples from dogs and cats presented to the Veterinary Teaching Hospital of Jordan University of Science and Technology between 2012 and 2024. Records from 232 clinical cases comprising 248 submitted samples were reviewed. Among 237 samples with known culture outcomes, 121 yielded bacterial growth (51.1%), resulting in 141 bacterial isolates. *Escherichia coli* (*E. coli*) (33.3%) was the most frequently recovered organism, followed by *Staphylococcus aureus* (*S. aureus*) (19.1%), *Staphylococcus* spp. (15.6%), and *Proteus* spp. (9.9%). AST results were available for 96/141 isolates (68.1%). Archived interpretations showed frequent non-susceptibility to several tested agents, whereas fluoroquinolone non-susceptibility was generally lower. Using the historical study rule of non-susceptibility to at least one agent in three or more antimicrobial classes, 87/96 isolates (90.6%) met the screening criterion. However, intermediate results were grouped with resistant results, AST panels and breakpoint standards varied over 13 years, and the archived records did not permit reliable exclusion of intrinsic resistance for every organism–drug combination. Accordingly, the MDR estimate is descriptive and should not be interpreted as a standardized contemporary prevalence. These data provide a baseline for companion-animal bacterial surveillance in Jordan and support prospective standardized AST and antimicrobial stewardship.

## Simple Summary

Bacterial infections are common in dogs and cats and are often treated with antimicrobials. Information on antimicrobial resistance (AMR) in companion animals remains limited in Jordan. This retrospective study examined laboratory records from dogs and cats presented to the Veterinary Teaching Hospital of Jordan University of Science and Technology between 2012 and 2024. The most frequently recovered bacteria were *Escherichia coli* (*E. coli*), *Staphylococcus aureus* (*S. aureus*), *Staphylococcus* spp., and *Proteus* spp. Non-susceptibility to several commonly tested antimicrobials was frequent among isolates with available antimicrobial susceptibility testing (AST) results. Because AST panels and interpretive standards varied during the 13-year period and archived records did not permit isolate-level exclusion of intrinsic resistance, the reported multidrug-resistance (MDR) classification should be interpreted as a retrospective screening estimate rather than a standardized contemporary MDR prevalence. The findings provide baseline information for companion-animal bacterial surveillance in Jordan and support standardized prospective AST and antimicrobial stewardship.

## 1. Introduction

Antimicrobial therapy is commonly used in dogs and cats for suspected or confirmed bacterial infections, particularly skin and soft-tissue, urinary, respiratory, and other infections [1,2,3]. Appropriate antimicrobial selection depends on clinical assessment and, when indicated, bacterial culture and antimicrobial susceptibility testing (AST). However, diagnostic testing is not consistently performed before treatment, and empirical prescribing remains common in companion-animal practice [4,5].

Antimicrobial resistance (AMR) can compromise treatment and has clinical and public-health relevance because companion animals may carry resistant bacteria shared with humans and the environment [6,7,8]. Surveillance based on standardized laboratory methods is therefore important for describing local resistance patterns, supporting antimicrobial stewardship, and identifying changes that warrant further investigation [9,10,11,12].

International studies have documented substantial geographic and temporal variation in bacterial distribution and AMR among companion animals, including *E. coli*, *Staphylococcus* spp., *Proteus* spp., *Pseudomonas* spp., and other clinically important organisms [13,14,15,16,17,18,19]. Differences among studies reflect host populations, sample types, referral status, antimicrobial exposure, laboratory methods, antimicrobial panels, and interpretive criteria, which should be considered when comparing resistance estimates [9,11,12,18].

In Jordan, AMR is well documented in human healthcare and livestock, and national policy recognizes the need for multisectoral AMR surveillance [20,21]. In contrast, companion-animal data remain scarce. A previous Jordanian study investigated methicillin-resistant *S. aureus* in dogs and associated personnel, but broader long-term surveillance of bacterial pathogens and antimicrobial susceptibility patterns in dogs and cats has not been reported [22]. This evidence gap limits the local baseline available for veterinary antimicrobial stewardship. Accordingly, this retrospective study aimed to characterize bacterial pathogens recovered from clinical samples from dogs and cats submitted to the Veterinary Teaching Hospital at Jordan University of Science and Technology (JUST), Jordan, between January 2012 and December 2024, and to describe the archived antimicrobial susceptibility and MDR screening patterns among isolates with available AST data.

## 2. Materials and Methods

### 2.1. Case Selection and Data Collection

A retrospective observational study was conducted using archived clinical and microbiological records from dogs and cats for which samples had been submitted for aerobic bacterial culture at the Veterinary Teaching Hospital, Jordan University of Science and Technology, between January 2012 and December 2024. The initial dataset contained 248 laboratory submissions from 232 clinical cases. Culture outcomes were known for 237 samples; 11 submissions had unavailable culture results and were retained only to describe the total submission denominator, but were excluded from calculations requiring a known culture outcome and from bacterial-isolate analyses. Samples with no bacterial growth were included in culture-outcome summaries. Culture-positive samples yielding more than one bacterial species contributed each recovered isolate to isolate-level analyses. The 232 cases represent clinical cases/laboratory episodes rather than a confirmed count of unique animals across the entire 13-year period because a permanent unique-animal identifier was not consistently available in the archived database.

Isolates without AST results were retained for bacterial-distribution analyses but excluded from antimicrobial susceptibility analyses. AST was performed as part of routine diagnostic service and was not available for every recovered isolate; the archived database did not consistently record why AST was not performed for individual isolates. Possible operational reasons cannot be reconstructed retrospectively and are therefore not assigned. Because the study was conducted at a referral veterinary teaching hospital and bacterial culture was requested according to clinical need rather than systematic surveillance, the relatively small number of submissions over 13 years reflects the available archived diagnostic records and should not be interpreted as the total burden of bacterial disease in the hospital population or Jordan small animal population.

Additionally, as this study was conducted at a referral veterinary teaching hospital, the study population may be affected by referral and selection bias, with possible overrepresentation of severe, chronic, recurrent, or treatment-refractory infections compared with the general companion-animal population. The dataset also did not contain a complete denominator of all dogs and cats examined at the hospital during the study period.

### 2.2. Sample Collection and Processing

Samples were aseptically collected by attending clinicians during diagnostic or therapeutic procedures following routine clinical procedures. Sample types included swabs (skin, wound, ear, vaginal, ocular), urine (collected via cystocentesis or catheterization), aspirates (body fluids and masses), and tissue biopsies. Standardized aseptic collection techniques were consistently applied throughout the study period to minimize contamination. Specimens were placed in appropriate transport media and submitted to the in-house diagnostic microbiology laboratory as soon as possible after collection, typically within 2 h.

### 2.3. Bacterial Culture and Identification

Samples were processed using routine conventional clinical microbiology procedures. Specimens were inoculated onto 5% sheep blood agar and MacConkey agar, with additional selective media used when indicated [23], and incubated aerobically at 35 °C ± 2 °C for 24–48 h. Identification was based on colony morphology, hemolysis, Gram staining, and a conventional biochemical algorithm using tests selected according to the presumptive organism, including catalase and coagulase testing for *staphylococci*, oxidase testing for oxidase-positive Gram-negative organisms, and indole and other routine biochemical reactions for enteric Gram-negative bacteria [23]. Commercial identification systems, MALDI-TOF MS, PCR, or sequencing were not routinely used during the study period. Isolates were reported at species level only when the archived laboratory identification supported that assignment; otherwise, they were retained at genus or phenotypic-group level. For *staphylococci*, coagulase-positive isolates identified by the historical laboratory workflow could be reported as *S. aureus*, whereas isolates not resolved to species were retained as *Staphylococcus* spp.; coagulase-negative isolates were reported separately. Because conventional phenotypic methods may misidentify closely related *staphylococci*, including *S. pseudintermedius*, species-level assignments are interpreted cautiously.

### 2.4. Antimicrobial Susceptibility Testing

Antimicrobial susceptibility testing was performed using the Kirby–Bauer disk-diffusion method on Mueller–Hinton agar [24]. The antimicrobial agents tested included gentamicin (10 µg), streptomycin (10 µg), neomycin (30 µg), ciprofloxacin (5 µg), enrofloxacin (5 µg), oxytetracycline (30 µg), doxycycline (30 µg), chlortetracycline (30 µg), tetracycline (30 µg), ampicillin (10 µg) or amoxicillin (10 µg), penicillin (10 units), cefquinome (30 µg), erythromycin (15 µg), and trimethoprim/sulfamethoxazole (1.25/23.75 µg), depending on laboratory availability and routine diagnostic practice. Although the specific CLSI editions and breakpoint criteria applied throughout 2012–2024 were not consistently documented in the archived records, zone diameters were interpreted according to the CLSI-based standards in use by the laboratory at the time of testing [25]. The archived records did not consistently preserve the exact CLSI edition, whether a veterinary species-specific breakpoint or a human-derived surrogate was applied to each organism–drug combination, or the raw zone diameter needed for retrospective reinterpretation under one contemporary standard. Consequently, historical S/I/R calls could not be harmonized retrospectively. For descriptive analysis, intermediate and resistant historical calls were combined and are therefore reported as non-susceptible rather than as resistant. This approach, together with changing AST panels, is a major limitation and precludes direct temporal comparison across the 13-year period.

For the historical MDR screening analysis, isolates with AST results for at least three antimicrobial classes were evaluated using the study rule of non-susceptibility to at least one agent in three or more classes, based on the framework of Magiorakos et al. [26]. However, that framework is intended for acquired resistance and requires intrinsic resistance to be excluded. The archived records did not preserve sufficient isolate-level breakpoint and intrinsic-resistance information to reconstruct a fully standardized acquired-resistance MDR classification for every organism–drug combination. In particular, organism–drug combinations with known or possible intrinsic non-susceptibility, such as tetracyclines in *Proteus* spp. and several nonstandard combinations, could have contributed to the historical classification. Therefore, the reported value is retained only as an apparent MDR/non-susceptibility screening estimate and not as a definitive standardized MDR prevalence. Most evaluated isolates had results spanning five to six antimicrobial classes; all 96 isolates included in this screening analysis had results for at least three classes.

### 2.5. Data and Statistical Analysis

Data were compiled and analyzed using Microsoft Excel (Microsoft 365, Microsoft Corporation, Redmond, WA, USA). Categorical variables were summarized as frequencies and percentages, and continuous variables as mean ± standard deviation when applicable. Culture positivity was calculated among samples with known culture outcomes (121/237), while the total number of submitted samples (*n* = 248) was reported separately. Exact 95% binomial confidence intervals were calculated for culture positivity, AST availability, and the overall apparent MDR screening proportion. Resistance/non-susceptibility percentages for each organism–antimicrobial combination used the number of isolates with an available result for that specific agent as the denominator. The study was designed as a descriptive retrospective analysis; formal inferential comparisons between dogs and cats or between Gram-positive and Gram-negative organisms were not performed because of sparse organism-specific strata, heterogeneous AST panels, and incomplete AST availability, which would make such comparisons difficult to interpret.

### 2.6. Data Handling and Quality Control

Data were reviewed for completeness and consistency prior to analysis. Records were included according to predefined inclusion, and missing demographic or clinical information did not result in exclusion when microbiological results were available. Duplicate records were reviewed to ensure accurate representation of clinical samples and bacterial isolates. Multiple bacterial isolates recovered from the same culture were retained for isolate-level analysis. Multiple samples collected from different anatomical sites during the same clinical episode were retained, whereas serial or repeat sampling of the same anatomical site during follow-up was not identified in the archived records.

### 2.7. Ethics Statement

This study was based on a retrospective analysis of anonymized clinical data and microbiological records from the Veterinary Teaching hospital, Jordan University of Science and Technology. No animals were enrolled specifically for this study, and no additional procedures or interventions were performed for research purposes. According to institutional policy, formal ethical approval was not required because the study involved the retrospective use of existing anonymized clinical records.

## 3. Results

### 3.1. Patient Characteristics and Clinical Presentation

The study included 232 clinical cases/laboratory episodes submitted for aerobic bacterial culture between January 2012 and December 2024, comprising 89 dog cases (38.36%) and 143 cat cases (61.64%) (Table 1). The mean age was 3.61 ± 3.13 years in dogs and 2.80 ± 2.52 years in cats. Sex information was available for 220 cases. Because the archived database did not consistently contain a permanent unique-animal identifier, the 232 records should not be interpreted as a verified count of unique animals over the 13-year period.

A total of 248 clinical samples were submitted from 232 clinical cases during the study period. Seven cases contributed more than one sample, including samples collected from different anatomical sites during the same clinical episode. Clinical presentations involved multiple body systems and differed between species (Table 2). Integumentary conditions were the most frequently recorded category, accounting for 61 cases (26.3%), including 24 dog cases (10.3%) and 37 cat cases (15.9%). Urinary tract presentations accounted for 42 cases (18.1%) and were more frequent among cats (34 cases; 14.7%) than dogs (8 cases; 3.4%). Reproductive tract presentations accounted for 17 cases (7.3%), while gastrointestinal tract and ear presentations each accounted for 15 cases (6.5%). Respiratory tract (10 cases; 4.3%), ocular (7 cases; 3.0%), musculoskeletal (4 cases; 1.7%), neurological (2 cases; 0.9%), and lymphatic (1 case; 0.4%) presentations were less frequent. Clinical information was unavailable or could not be assigned to a specific body system in 42 cases (18.1%).

### 3.2. Culture Outcomes and Bacterial Isolates

Out of 248 submitted samples, 121 (48.8%) yielded positive bacterial growth, 116 (46.8%) yielded no bacterial growth, and culture results were unavailable for 11 (4.4%). Among the 237 samples with known culture outcomes, culture positivity was 51.1% (121/237; 95% CI, 44.5–57.6%). Among the 232 clinical cases, seven (3.0%) contributed multiple samples collected during the same clinical episode. Among the 121 culture-positive samples, 11 (9.1%) yielded polymicrobial growth. Overall, the 121 positive cultures resulted in the recovery of 141 bacterial isolates.

### 3.3. Distribution of Bacterial Isolates

A total of 141 bacterial isolates were recovered from the 121 culture-positive samples during the study period (Table 3). Of these, 57 (40.4%) were recovered from dogs and 84 (59.6%) from cats. *E. coli* was the most frequently recovered organism overall (47/141, 33.3%), followed by *S. aureus* (27/141, 19.1%), *Staphylococcus* spp. (22/141, 15.6%), and *Proteus* spp. (14/141, 9.9%).

Among the 57 isolates recovered from dogs, *E. coli* was the most frequent (12/57, 21.1%), followed by *S. aureus* (10/57, 17.5%) and *Proteus* spp. (8/57, 14.0%). *Staphylococcus* spp. and coagulase-negative staphylococci each accounted for 7/57 isolates (12.3%), while *Pseudomonas* spp. accounted for 5/57 (8.8%). The remaining dog isolates comprised alpha-hemolytic streptococci (2/57, 3.5%), Group G β-hemolytic streptococci (2/57, 3.5%), *Pasteurella* spp. (2/57, 3.5%), *Bacillus* spp. (1/57, 1.8%), and *Klebsiella* spp. (1/57, 1.8%).

Among the 84 isolates recovered from cats, *E. coli* predominated (35/84, 41.7%), followed by *S. aureus* (17/84, 20.2%), *Staphylococcus* spp. (15/84, 17.9%), and *Proteus* spp. (6/84, 7.1%). Less frequently recovered organisms included alpha-hemolytic streptococci (3/84, 3.6%), coagulase-negative staphylococci (2/84, 2.4%), *Pseudomonas* spp. (2/84, 2.4%), *Bacillus* spp. (2/84, 2.4%), *Enterococcus* spp. (1/84, 1.2%), and *Streptococcus* spp. (1/84, 1.2%). No Group G β-hemolytic streptococci, *Pasteurella* spp., or *Klebsiella* spp. were recovered from cats.

### 3.4. Antimicrobial Susceptibility Patterns

AST results were available for 96 of 141 bacterial isolates (68.1%; 95% CI, 60.0–75.7%), while AST results were unavailable for 45 isolates (31.9%). Detailed organism-specific results are provided in Supplementary Table S1. Among *E. coli* isolates, the highest non-susceptibility frequencies were recorded for penicillin (23/23, 100%), erythromycin (17/18, 94.4%), and streptomycin (21/23, 91.3%). Lower frequencies were observed for ciprofloxacin and enrofloxacin (4/30, 13.3% each) and chlortetracycline (1/9, 11.1%). Among *S. aureus* isolates, non-susceptibility was highest for trimethoprim–sulfamethoxazole (20/22, 90.9%), penicillin (19/21, 90.5%), and streptomycin (18/20, 90.0%). Non-susceptibility to ciprofloxacin and enrofloxacin was lower, with 6/22 isolates (27.3%) non-susceptible to each agent. Among *Staphylococcus* spp., non-susceptibility was recorded most frequently for streptomycin (10/12, 83.3%), ampicillin (14/17, 82.4%), erythromycin (9/11, 81.8%), and trimethoprim–sulfamethoxazole (12/16, 75.0%). Lower frequencies were observed for ciprofloxacin and enrofloxacin (2/17, 11.8% each). Among *Proteus* spp., non-susceptibility was frequent for several tested antimicrobial agents, while enrofloxacin showed a comparatively lower frequency of non-susceptibility (2/9, 22.2%).

### 3.5. Multidrug Resistance Screening

Among the 141 bacterial isolates recovered during the study period, 96 had available AST results and were tested against at least three antimicrobial classes, making them eligible for MDR screening (Table 4). Of these, 87/96 (90.6%; 95% CI, 82.9–95.6%) met the study MDR screening criterion, while 9/96 (9.4%) did not. The MDR screening criterion was met by 26/30 (86.7%) *E. coli*, 20/22 (90.9%) *S. aureus*, and 15/17 (88.2%) *Staphylococcus* spp. isolates. All evaluated coagulase-negative staphylococci (6/6), *Proteus* spp. (9/9), and *Pseudomonas* spp. (6/6) met the MDR screening criterion. Among less frequently evaluated organisms, the MDR screening criterion was met by 2/2 α-hemolytic streptococci and by the single evaluated isolates of Group G β-hemolytic streptococci, *Klebsiella* spp., and *Streptococcus* spp.; the single evaluated *Bacillus* spp. isolate did not meet the criterion. *Pasteurella* spp. and *Enterococcus* spp. could not be evaluated because AST results were unavailable. Estimates based on one or two isolates should be interpreted cautiously because of the small denominators.

## 4. Discussion

### 4.1. Bacterial Distribution in Companion Animals

This study provides a descriptive baseline of bacterial isolates and archived antimicrobial susceptibility results from companion-animal clinical samples submitted to a referral veterinary teaching hospital in Jordan between 2012 and 2024. Interpretation is intentionally limited to the available retrospective laboratory data; the study was not designed to estimate population prevalence, infer transmission, or evaluate temporal trends.

Integumentary and urinary tract presentations were common among submitted samples, consistent with the types of infections for which bacterial culture is frequently requested in companion animals [3,18,27,28,29]. However, organism-by-anatomical-site cross-tabulation was not available in a form suitable for analysis, and 18.1% of submissions had an undocumented or unassignable site. Therefore, the distribution of particular organisms cannot be attributed to specific body systems from these data [30]. Among samples with known culture outcomes, 51.1% yielded bacterial growth. Culture-negative results may occur for several reasons, including prior antimicrobial exposure, low bacterial load, sample quality, noninfectious disease, or organisms not recovered by routine aerobic culture [5,31]. The retrospective database did not contain the information needed to determine the reason for individual negative cultures. *E. coli* was the most frequently recovered organism, followed by *S. aureus*, *Staphylococcus* spp., and *Proteus* spp. Similar organisms are commonly reported in companion-animal diagnostic surveillance [13,14,15,16,18,28,29,30,31,32]. Cross-study differences in their relative frequencies should be interpreted cautiously because referral populations, sample composition, laboratory methods, and reporting practices differ.

The relatively frequent historical identification of *S. aureus* requires particular caution. *S. pseudintermedius* is a major canine staphylococcal commensal and pathogen, and conventional phenotypic methods may not reliably distinguish closely related coagulase-positive staphylococci [30,33]. Because MALDI-TOF MS or molecular confirmation was not routinely available, some species-level assignments may have been misclassified. The present data therefore cannot support conclusions regarding human-to-animal transmission of *S. aureus*; molecular typing and paired human–animal sampling would be required to investigate transmission [22].

Cats contributed more submissions and isolates than dogs, but the dataset does not provide the hospital population denominator or information needed to distinguish effects of local pet demographics, referral patterns, healthcare-seeking behavior, or case mix. The species distribution should therefore be interpreted as a characteristic of the submitted diagnostic records rather than as a population-level difference.

### 4.2. Antimicrobial Resistance Patterns

Archived AST results showed frequent non-susceptibility to several tested antimicrobial classes. Direct comparison with other studies is limited by differences in bacterial populations, AST panels, breakpoint standards, and referral status [8,10,14,15,17,18,30,32]. In the present dataset, AST panels changed over time and the exact breakpoint edition and organism-specific interpretive source were not consistently preserved; therefore, the historical results cannot be reinterpreted under one contemporary standard. For *E. coli* and staphylococci, lower archived non-susceptibility frequencies were generally observed for fluoroquinolones than for several older agents, but these findings should not be used to support empirical fluoroquinolone selection. Fluoroquinolones are critically important antimicrobials, and treatment decisions should be guided by appropriate culture and AST whenever indicated [12].

*Proteus* spp. illustrate an important limitation of retrospective resistance summaries. This genus has recognized intrinsic resistance to tetracyclines, and intrinsic resistance should not be interpreted as acquired resistance or counted toward a standardized MDR definition [26,27]. Accordingly, the tetracycline results are retained only as historical laboratory observations and are not used to infer acquired resistance.

Methicillin resistance in *staphylococci* could not be assessed reliably because the archived dataset did not consistently contain oxacillin/cefoxitin testing or mecA/mecC confirmation. Consequently, the absence of explicitly recorded MRSA or MRSP is a limitation of the testing and identification data rather than evidence of absence.

### 4.3. Multidrug Resistance

The historical screening rule classified 87/96 isolates (90.6%) as apparent MDR/non-susceptible to at least one agent in three or more antimicrobial classes. However, this estimate is constrained by the retrospective nature of the dataset because the internationally accepted MDR framework concerns acquired resistance and requires exclusion of intrinsic resistance [26], which could not be consistently determined from the archived isolate-level information. In addition, grouping intermediate with resistant results and variation in AST panels and interpretive criteria across the 13-year study period may have influenced the estimated proportion. Accordingly, the 90.6% value should be interpreted as a descriptive screening estimate derived from archived laboratory records rather than as a standardized MDR prevalence, and comparisons with estimates reported in other studies should be made cautiously [15,17,18,30]. Referral-case selection may also have influenced the observed resistance burden because referral hospitals may receive complicated or previously treated infections [15,17,30,34]; however, previous antimicrobial treatment histories were unavailable and this possibility could not be evaluated directly. Species-specific estimates based on only one or two isolates should likewise be interpreted cautiously because of the small denominators. Larger prospective studies using standardized organism-specific breakpoints, consistent AST panels, explicit antimicrobial-class assignments, and exclusion of intrinsic resistance are needed to provide robust species-specific MDR estimates and permit temporal comparisons. These findings support routine bacteriological culture and standardized AST in companion-animal practice and provide baseline data for prospective surveillance, although their interpretation is primarily descriptive rather than causal or temporal.

### 4.4. Study Limitations

The present study has several limitations. It was a retrospective analysis of diagnostic records from a referral veterinary teaching hospital, and the relatively small number of archived submissions over 13 years reflects clinically requested cultures rather than systematic surveillance. The dataset did not provide the denominator of all hospital patients, and the 232 records represent clinical cases/laboratory episodes rather than a verified number of unique animals. Consequently, the findings may not be representative of the broader companion-animal population in Jordan.

Previous antimicrobial treatment, underlying disease, and other host-level risk factors were not consistently available. Some culture outcomes and AST results were missing, and AST was available for only 96/141 isolates. The reason AST was unavailable for individual isolates could not be reconstructed. Several organism-specific estimates were based on very small denominators and should be interpreted descriptively.

Bacterial identification relied primarily on conventional phenotypic and biochemical methods. Commercial identification systems, MALDI-TOF MS, and molecular confirmation were not routinely used; therefore, species-level misidentification is possible, particularly among closely related staphylococci such as *S. pseudintermedius*. Methicillin resistance could not be assessed reliably from the archived data.

AST panels and interpretive standards changed during the 13-year period, and the archived records did not consistently preserve the exact CLSI edition, breakpoint source, raw zone diameters, or isolate-level information required to exclude intrinsic resistance for every organism–drug combination [25,26]. Intermediate results were grouped with resistant results in the historical analysis. These limitations precluded retrospective reinterpretation under a single contemporary breakpoint standard, robust temporal analysis, and definitive standardized MDR classification. The study also lacked molecular resistance data and paired human–animal sampling. Future prospective surveillance should use standardized species identification, fixed or harmonized AST panels, explicit veterinary breakpoints, separate S/I/R reporting, exclusion of intrinsic resistance from MDR classification, and sufficient annual sample sizes for temporal analysis.

## 5. Conclusions

This retrospective study describes bacterial isolates and archived antimicrobial susceptibility results from companion-animal clinical samples submitted to a veterinary teaching hospital in Jordan during 2012–2024. *Escherichia coli*, *Staphylococcus aureus*, *Staphylococcus* spp., and *Proteus* spp. were the most frequently reported bacterial groups. Historical AST records showed frequent non-susceptibility to several tested agents, but interpretation is limited by incomplete AST availability, changing panels and breakpoints, possible species-level misidentification, grouping of intermediate with resistant results, and inability to exclude intrinsic resistance consistently.

The apparent MDR screening proportion should therefore be interpreted as a descriptive feature of the archived referral-hospital dataset rather than a standardized contemporary MDR prevalence. The findings provide baseline data for companion-animal bacterial surveillance in Jordan and support prospective surveillance using standardized identification, AST, breakpoint interpretation, and antimicrobial-stewardship procedures.

## Figures and Tables

**Table 1:** Demographic characteristics of dogs and cats included in the study.

Body System	Dog Cases, *n* (%)	Cat Cases, *n* (%)	Total
Clinical cases	89 (38.36)	143 (61.64)	232
Male	42 (47.19)	68 (47.55)	110
Female	43 (48.31)	67 (46.85)	110
Missing sex	4 (4.5)	8 (5.6)	12
Mean age ± SD (years)	3.61 ± 3.13	2.80 ± 2.52	-

**Table 2:** Distribution of clinical cases and submitted samples according to body system and host species.

Body System	Dogs, *n* (%)	Cats, *n* (%)	Total Cases, *n* (%)	Dog Submitted Samples, *n* (%)	Cat Submitted Samples, *n* (%)	Total Submitted Samples, *n* (%)
Integumentary	24 (10.3)	37 (15.9)	61 (26.3)	26 (10.5)	40 (16.1)	66 (26.6)
Urinary tract	8 (3.4)	34 (14.7)	42 (18.1)	9 (3.6)	34 (13.7)	43 (17.3)
Unknown	20 (8.6)	22 (9.5)	42 (18.1)	21 (8.5)	22 (8.9)	43 (17.3)
Reproductive tract	7 (3.0)	10 (4.3)	17 (7.3)	9 (3.6)	14 (5.6)	23 (9.3)
Gastrointestinal tract	4 (1.7)	11 (4.7)	15 (6.5)	5 (2.0)	12 (4.8)	17 (6.9)
Ear	7 (3.0)	8 (3.4)	15 (6.5)	7 (2.8)	8 (3.2)	15 (6.0)
Respiratory tract	4 (1.7)	6 (2.6)	10 (4.3)	4 (1.6)	6 (2.4)	10 (4.0)
Eye	4 (1.7)	3 (1.3)	7 (3.0)	4 (1.6)	4 (1.6)	8 (3.2)
Musculoskeletal system	3 (1.3)	1 (0.4)	4 (1.7)	3 (1.2)	1 (0.4)	4 (1.6)
Neurological system	1 (0.4)	1 (0.4)	2 (0.9)	1 (0.4)	1 (0.4)	2 (0.8)
Lymphatic system	1 (0.4)	0 (0.0)	1 (0.4)	1 (0.4)	0 (0.0)	1 (0.4)

Footnote: Percentages in the Dogs, Cats, and Total cases columns were calculated using the total number of clinical cases (*n* = 232) as the denominator. Percentages in the Dog submitted samples, Cat submitted samples, and Total submitted samples columns were calculated using the total number of submitted samples (*n* = 248) as the denominator. Therefore, percentages in the species-specific columns represent the proportion of the overall dataset contributed by each species and are not within-species percentages.

**Table 3:** Distribution of bacterial isolates recovered from clinical samples submitted from dogs and cats between 2012 and 2024.

Organism	Dog Isolates, *n* (%)	Cat Isolates, *n* (%)	Total Isolates, *n* (%)
*Escherichia coli*	12 (21.1)	35 (41.7)	47 (33.3)
*Staphylococcus aureus*	10 (17.5)	17 (20.2)	27 (19.1)
*Staphylococcus* spp.	7 (12.3)	15 (17.9)	22 (15.6)
*Proteus* spp.	8 (14.0)	6 (7.1)	14 (9.9)
Coagulase-negative staphylococci	7 (12.3)	2 (2.4)	9 (6.4)
*Pseudomonas* spp.	5 (8.8)	2 (2.4)	7 (5.0)
Alpha-hemolytic streptococci	2 (3.5)	3 (3.6)	5 (3.5)
*Bacillus* spp.	1 (1.8)	2 (2.4)	3 (2.1)
Group G β-hemolytic streptococci	2 (3.5)	0 (0.0)	2 (1.4)
*Pasteurella* spp.	2 (3.5)	0 (0.0)	2 (1.4)
*Enterococcus* spp.	0 (0.0)	1 (1.2)	1 (0.7)
*Klebsiella* spp.	1 (1.8)	0 (0.0)	1 (0.7)
*Streptococcus* spp.	0 (0.0)	1 (1.2)	1 (0.7)
Total	57 (100)	84 (100)	141 (100)

**Table 4:** Multidrug resistance screening among bacterial isolates recovered from dogs and cats between 2012 and 2024.

Organism	Total Isolates (*n*)	AST Available (*n*)	Tested ≥ 3 Classes (*n*)	MDR Isolates (*n*)	Non-MDR Isolates (*n*)	MDR (%)
*Escherichia coli*	47	30	30	26	4	86.7
*Staphylococcus aureus*	27	22	22	20	2	90.9
*Staphylococcus* spp.	22	17	17	15	2	88.2
Coagulase-negative *staphylococci*	9	6	6	6	0	100.0
*Proteus* spp.	14	9	9	9	0	100.0
*Pseudomonas* spp.	7	6	6	6	0	100.0
α-hemolytic *streptococci*	5	2	2	2	0	100.0
*Bacillus* spp.	3	1	1	0	1	0.0
Group G β-hemolytic *streptococci*	2	1	1	1	0	100.0
*Pasteurella* spp.	2	0	0	0	0	–
*Enterococcus* spp.	1	0	0	0	0	–
*Klebsiella* spp.	1	1	1	1	0	100.0
*Streptococcus* spp.	1	1	1	1	0	100.0
Total	141	96	96 *	87	9	90.6

* All isolates with available antimicrobial susceptibility testing (AST) results (*n* = 96) were tested against ≥3 antimicrobial classes and were included in the MDR screening analysis. Percentages meeting the MDR screening criterion were calculated using the number of isolates eligible for MDR screening within each organism as the denominator. The overall percentage meeting the MDR screening criterion was calculated using all isolates eligible for MDR screening (*n* = 96) as the denominator. Percentages based on one or two evaluated isolates are descriptive only and should be interpreted cautiously because the small denominators limit their reliability and generalizability. – Not applicable because no isolates were available for MDR screening.

## Data Availability

The data presented in this study are available on request from the corresponding author. The data are not publicly available because they are derived from clinical records and access is restricted to protect clinical-record confidentiality.

## Supplementary Materials

The following supporting information can be downloaded at: https://www.mdpi.com/article/10.3390/vetsci13090892/s1, Table S1: Antimicrobial resistance profile of bacterial isolates recovered from dogs and cats between 2012 and 2024.

## Abbreviations

The following abbreviations are used in this manuscript:

MDR	Multidrug resistant
AST	Antimicrobial susceptibility testing
