# Supplementary material for: Antimicrobial Susceptibility and Apparent Multidrug Resistance Screening Patterns of Bacterial Pathogens Recovered from Clinical Samples of Companion Animals in Jordan, 2012–2024: A Retrospective Study from a Veterinary Teaching Hospital
Source: Vet Sci. 2026 Aug 31;13(9):892. doi: 10.3390/vetsci13090892 (PMC13611876; doi:10.3390/vetsci13090892)
Supplement: Supplementary file 1 [file vetsci-13-00892-s001.zip › vetsci-4471615-supplementary.pdf]

Table S1. Antimicrobial resistance profile of bacterial isolates recovered from dogs and cats between 2012 and 2024.

Organism	Total Isolates	Antimicrobial	Tested isolates (n)	Resistant isolates (n)*	Resistance %
**Escherichia coli**	47	Gentamicin	30	18	60.00
		Streptomycin	23	21	91.30
		Neomycin	4	4	100.00
		Ciprofloxacin	30	4	13.33
		Enrofloxacin	30	4	13.33
		Oxytetracycline	21	17	80.95
		Doxycycline	23	13	56.52
		Chlortetracycline	9	1	11.11
		Tetracycline	6	4	66.67
		Ampicillin	27	22	81.48
		Amoxicillin	28	25	89.29
		Penicillin	23	23	100.00
		Cefquinome	0	-	-
		Erythromycin	18	17	94.44
		Trimethoprim/sulfamethoxazole	27	17	62.96
**Pseudomonas spp.**	7	Gentamicin	6	3	50.00
		Streptomycin	6	6	100.00
		Neomycin	0	-	-
		Ciprofloxacin	6	2	33.33
		Enrofloxacin	6	4	66.67
		Oxytetracycline	5	5	100.00
		Doxycycline	4	4	100.00
		Chlortetracycline	1	1	100.00
		Tetracycline	1	1	100.00
		Ampicillin	6	5	83.33
		Amoxicillin	6	5	83.33
		Penicillin	6	5	83.33
		Cefquinome	0	-	-
		Erythromycin	5	5	100.00
		Trimethoprim/sulfamethoxazole	6	6	100.00
**Group G β-hemolytic streptococci**	2	Gentamicin	0	-	-
		Streptomycin	1	1	100.00
		Neomycin	0	-	-
		Ciprofloxacin	1	0	0.00
		Enrofloxacin	1	0	0.00
		Oxytetracycline	1	1	100.00
		Doxycycline	1	1	100.00
		Chlortetracycline	0	-	-
		Tetracycline	0	-	-
		Ampicillin	1	0	0.00

		Amoxicillin	1	0	0.00
		Penicillin	1	1	100.00
		Cefquinome	0	-	-
		Erythromycin	1	1	100.00
		Trimethoprim/sulfamethoxazole	1	1	100.00
**Staphylococcus spp.**	22	Gentamicin	17	10	58.82
		Streptomycin	12	10	83.33
		Neomycin	5	5	100.00
		Ciprofloxacin	17	2	11.76
		Enrofloxacin	17	2	11.76
		Oxytetracycline	5	2	40.00
		Doxycycline	12	6	50.00
		Chlortetracycline	3	0	0.00
		Tetracycline	11	8	72.73
		Ampicillin	17	14	82.35
		Amoxicillin	17	13	76.47
		Penicillin	12	9	75.00
		Cefquinome	0	-	-
		Erythromycin	11	9	81.82
		Trimethoprim/sulfamethoxazole	16	12	75.00
**Bacillus spp.**	3	Gentamicin	1	0	0.00
		Streptomycin	1	0	0.00
		Neomycin	0		
		Ciprofloxacin	1	0	0.00
		Enrofloxacin	1	0	0.00
		Oxytetracycline	1	0	0.00
		Doxycycline	0	-	-
		Chlortetracycline	0	-	-
		Tetracycline	0	-	-
		Ampicillin	1	0	0.00
		Amoxicillin	1	0	0.00
		Penicillin	1	0	0.00
		Cefquinome	0	-	-
		Erythromycin	1	0	0.00
		Trimethoprim/sulfamethoxazole	1	0	0.00
**Proteus spp.**	14	Gentamicin	9	5	55.56
		Streptomycin	9	7	77.78
		Neomycin	0	-	-
		Ciprofloxacin	9	4	44.44
		Enrofloxacin	9	2	22.22
		Oxytetracycline	9	9	100.00
		Doxycycline	6	6	100.00
		Chlortetracycline	1	1	100.00
		Tetracycline	0	-	-
		Ampicillin	9	9	100.00

		Amoxicillin	9	9	100.00
		Penicillin	8	8	100.00
		Cefquinome	0	-	-
		Erythromycin	8	8	100.00
		Trimethoprim/sulfamethoxazole	9	9	100.00
**Enterococcus spp.**	1	Gentamicin	0	-	-
		Streptomycin	0	-	-
		Neomycin	0	-	-
		Ciprofloxacin	0	-	-
		Enrofloxacin	0	-	-
		Oxytetracycline	0	-	-
		Doxycycline	0	-	-
		Chlortetracycline	0	-	-
		Tetracycline	0	-	-
		Ampicillin	0	-	-
		Amoxicillin	0	-	-
		Penicillin	0	-	-
		Cefquinome	0	-	-
		Erythromycin	0	-	-
		Trimethoprim/sulfamethoxazole	0	-	-
**Coagulase-negative staphylococci**	9	Gentamicin	6	3	50.00
		Streptomycin	5	5	100.00
		Neomycin	0	-	-
		Ciprofloxacin	6	1	16.67
		Enrofloxacin	5	1	20.00
		Oxytetracycline	6	6	100.00
		Doxycycline	6	6	100.00
		Chlortetracycline	1	1	100.00
		Tetracycline	0	-	-
		Ampicillin	6	5	83.33
		Amoxicillin	6	5	83.33
		Penicillin	6	5	83.33
		Cefquinome	0	-	-
		Erythromycin	5	5	100.00
		Trimethoprim/sulfamethoxazole	6	5	83.33
**Staphylococcus aureus**	27	Gentamicin	22	10	45.45
		Streptomycin	20	18	90.00
		Neomycin	1	1	100.00
		Ciprofloxacin	22	6	27.27
		Enrofloxacin	22	6	27.27
		Oxytetracycline	21	15	71.43
		Doxycycline	18	13	72.22
		Chlortetracycline	5	3	60.00
		Tetracycline	1	0	0.00

		Ampicillin	21	16	76.19
		Amoxicillin	22	17	77.27
		Penicillin	21	19	90.48
		Cefquinome	1	1	100.00
		Erythromycin	17	15	88.24
		Trimethoprim/sulfamethoxazole	22	20	90.91
**Alpha-hemolytic streptococci**	5	Gentamicin	2	2	100.00
		Streptomycin	2	2	100.00
		Neomycin	0		
		Ciprofloxacin	2	1	50.00
		Enrofloxacin	2	1	50.00
		Oxytetracycline	2	2	100.00
		Doxycycline	1	1	100.00
		Chlortetracycline	0	-	-
		Tetracycline	0	-	-
		Ampicillin	1	1	100.00
		Amoxicillin	2	1	50.00
		Penicillin	2	1	50.00
		Cefquinome	0		
		Erythromycin	2	2	100.00
		Trimethoprim/sulfamethoxazole	2	2	100.00
**Pasteurella spp.**	2	Gentamicin	0	-	-
		Streptomycin	0	-	-
		Neomycin	0	-	-
		Ciprofloxacin	0	-	-
		Enrofloxacin	0	-	-
		Oxytetracycline	0	-	-
		Doxycycline	0	-	-
		Chlortetracycline	0	-	-
		Tetracycline	0	-	-
		Ampicillin	0	-	-
		Amoxicillin	0	-	-
		Penicillin	0	-	-
		Cefquinome	0	-	-
		Erythromycin	0	-	-
		Trimethoprim/sulfamethoxazole	0	-	-
**Klebsiella spp.**	1	Gentamicin	1	0	0.00
		Streptomycin	1	1	100.00
		Neomycin	0	-	-
		Ciprofloxacin	1	0	0.00
		Enrofloxacin	1	0	0.00
		Oxytetracycline	1	0	0.00
		Doxycycline	0	-	-
		Chlortetracycline	0	-	-
		Tetracycline	0	-	-

		Ampicillin	1	1	100.00
		Amoxicillin	1	1	100.00
		Penicillin	1	1	100.00
		Cefquinome	0	-	-
		Erythromycin	1	1	100.00
		Trimethoprim/sulfamethoxazole	1	1	100.00
**Streptococcus spp.**	1	Gentamicin	1	0	0.00
		Streptomycin	1	1	100.00
		Neomycin	0	-	-
		Ciprofloxacin	1	0	0.00
		Enrofloxacin	1	0	0.00
		Oxytetracycline	1	1	100.00
		Doxycycline	1	0	0.00
		Chlortetracycline	0	-	-
		Tetracycline	0	-	-
		Ampicillin	1	1	100.00
		Amoxicillin	1	1	100.00
		Penicillin	1	1	100.00
		Cefquinome	0	-	-
		Erythromycin	1	1	100.00
		Trimethoprim/sulfamethoxazole	1	1	100.00

- Resistance percentages were calculated using the number of isolates tested against each antimicrobial agent as the denominator.
- - indicate that the antimicrobial agent was not tested for that organism.
- Intermediate isolates were classified as resistant for analytical purposes.
- Percentages based on one or two tested isolates are descriptive only and should be interpreted with caution because the small denominators limit the reliability and generalizability of these estimates.
